# A Worldwide Competition to Compare the Speed and Chemotactic Accuracy of Neutrophil-Like Cells

**DOI:** 10.1371/journal.pone.0154491

**Published:** 2016-06-22

**Authors:** Monica Skoge, Elisabeth Wong, Bashar Hamza, Albert Bae, Joseph Martel, Rama Kataria, Ineke Keizer-Gunnink, Arjan Kortholt, Peter J. M. Van Haastert, Guillaume Charras, Christopher Janetopoulos, Daniel Irimia

**Affiliations:** 1 Joseph Henry Laboratories of Physics and Lewis-Sigler Institute for Integrative Genomics, Princeton University, Princeton, New Jersey, United States of America; 2 BioMEMS Resource Center, Massachusetts General Hospital, Shriners Burns Hospital, Harvard Medical School, Boston, Massachusetts, United States of America; 3 Max Planck Institute for Dynamics and Self Organization, Göttingen, Germany; 4 Department of Biomedical Engineering, Wentworth Institute of Technology, Boston, Massachusetts, United States of America; 5 Department of Cell Biochemistry, University of Groningen, Groningen, Netherlands; 6 London Centre for Nanotechnology and Department of Cell and Developmental Biology, University College London, London, United Kingdom; 7 University of the Sciences, Philadelphia, Pennsylvania, United States of America; Federal Institute for Vaccines and Biomedicines, GERMANY

## Abstract

Chemotaxis is the ability to migrate towards the source of chemical gradients. It underlies the ability of neutrophils and other immune cells to hone in on their targets and defend against invading pathogens. Given the importance of neutrophil migration to health and disease, it is crucial to understand the basic mechanisms controlling chemotaxis so that strategies can be developed to modulate cell migration in clinical settings. Because of the complexity of human genetics, *Dictyostelium* and HL60 cells have long served as models system for studying chemotaxis. Since many of our current insights into chemotaxis have been gained from these two model systems, we decided to compare them side by side in a set of winner-take-all races, the Dicty World Races. These worldwide competitions challenge researchers to genetically engineer and pharmacologically enhance the model systems to compete in microfluidic racecourses. These races bring together technological innovations in genetic engineering and precision measurement of cell motility. Fourteen teams participated in the inaugural Dicty World Race 2014 and contributed cell lines, which they tuned for enhanced speed and chemotactic accuracy. The race enabled large-scale analyses of chemotaxis in complex environments and revealed an intriguing balance of speed and accuracy of the model cell lines. The successes of the first race validated the concept of using fun-spirited competition to gain insights into the complex mechanisms controlling chemotaxis, while the challenges of the first race will guide further technological development and planning of future events.

## Introduction

Neutrophils are our first line of defense against invading pathogens. They are recruited to the site of wounds, kill bacteria and fungi via various mechanisms [1] and signal via cytokines to help coordinate the immune response [2, 3]. Crucially, these defense mechanisms are only effective in warding off infection if neutrophils are able to move swiftly and accurately to the site of the wound in the first place. Indeed, in clinical settings where neutrophil motility and chemotaxis are impaired, patients are at a high risk for infection [4, 5]. In other conditions, overzealous neutrophilic infiltration can unnecessarily damage normal tissues [6, 7] and impair organ function, e.g. in acute respiratory distress syndrome [8], arthritis [9], ischemia-reperfusion injury [10], or aging [11]. Despite the clear importance of neutrophil migration in many diseases, little is known about how to enhance or inhibit migration for therapeutic use in alleviating many of these conditions [12].

Neutrophils and other immune cells crawl in a manner very similar to amoeboid protozoa, by coordinated protrusions and retractions of a dynamic cytoskeleton. Immune cells and amoeba also share similar mechanisms of steering their motion up or down chemical gradients in a process called chemotaxis. The social amoeba *Dictyostelium discoideum* (Dicty) has proven a valuable and genetically tractable model system for understanding the fundamental mechanisms of neutrophil motility and chemotaxis [13, 14]. An equally important model system is the human promyelocytic cell line, HL60, which differentiates into neutrophils following treatment with dimethyl sulfoxide [15–17]. Decades of research in these systems have led to the discovery of many of the molecular components of the chemotaxis network and have shown that they are surprisingly well conserved between *D*. *discoideum* and humans [18]. While much has been learned about how to disrupt chemotaxis in these model systems [19], less is known about how to enhance it. Moreover, how the molecular components interact to give rise to cellular behaviors is complex [20] and integrating the results of different mutant studies to create a predictive model of amoeboid chemotaxis remains challenging, underlying the need for collaborative, larger-scale studies [21]. Finally, little is known about how to connect the behavior of cells in simple chemotaxis assays to the optimal performance of neutrophils fighting infection in complex *in vivo* environments.

Towards the broad goal of enhancing neutrophil migration in conditions of disease by building on fundamental research in model systems, we started a worldwide competition, the Dicty World Race. This competition challenged Dicty and HL60 researchers to apply their knowledge of chemotaxis to engineer the “ultimate” migrating cells to compete in a maze-like racecourse, which mimics the natural environment neutrophils move in. Unlike typical athletic competitions, genetic engineering and chemical “doping” were not only allowed, but were highly encouraged. Researchers accustomed to working with simple chemical gradients had to envision how they could optimize chemotaxis in a complex racecourse and tried a variety of strategies, *e*.*g*. manipulating adhesion, polarity, sensitivity and speed. In addition to being fun and fostering a friendly rivalry among researchers from several countries, the race enabled a large-scale comparison of motility and chemotaxis in the engineered cell lines, allowing exploration of a diverse set of strategies for enhancing chemotactic performance. Moreover, the race enabled a side-by-side comparison of the model systems and revealed intriguing and unexpected differences in behavior. In what follows, we present (i) the results of the first Dicty World Race 2014, (ii) an analysis of cell trajectories characterizing differences in motility and chemotaxis between Dicty and HL60 cells and exploring potential tradeoffs between speed and chemotactic accuracy, and (iii) the successes and pitfalls of the 2014 race along with suggestions for overcoming these challenges in future races.

## Materials and Methods

### Fabrication

Devices were fabricated in the cleanroom at the BioMEMS Resource Center, using standard photolithography and soft-lithography techniques. First, a master four-inch silicon wafer (Desert Silicon, Grandale, AZ) was spin-coated with SU-8 5 (thickness ~5 μm, Microchem, Newton, MA) to produce the first layer of the design, representing the migration channels. A second layer of SU-8 100 photoresist was then spun and baked, following the standard protocol as recommended by the manufacturer, to define the central and chemoattractant chambers (thickness ~120 μm). The microscale photo-patterned features were then employed to make channels in polydimethylsiloxane (PDMS, Sylgard, 184, Elsworth Adhesives, Wilmington, MA). Briefly, the PDMS base and curing agent were mixed (10:1 ratio) and poured on the master and gas bubbles were removed in a vacuum chamber. After baking for 12 hours at 80° C, the PDMS layer covering the master was peeled off, punched first with a 1.5 mm puncher (Harris Uni-Core, Ted Pella Inc., Reading, CA) to define the inlet to the device and then with a 5 mm puncher to release the device from the PDMS slab. Donut-shaped devices were then exposed to 35 seconds of oxygen plasma along with a 24-well, glass-bottom plate (MatTek Co., Ashland, MA). The PDMS devices were then bonded to the glass-bottom, multi-well plate and baked at 70°C for 15 minutes.

### Device design

**Fig 1:**
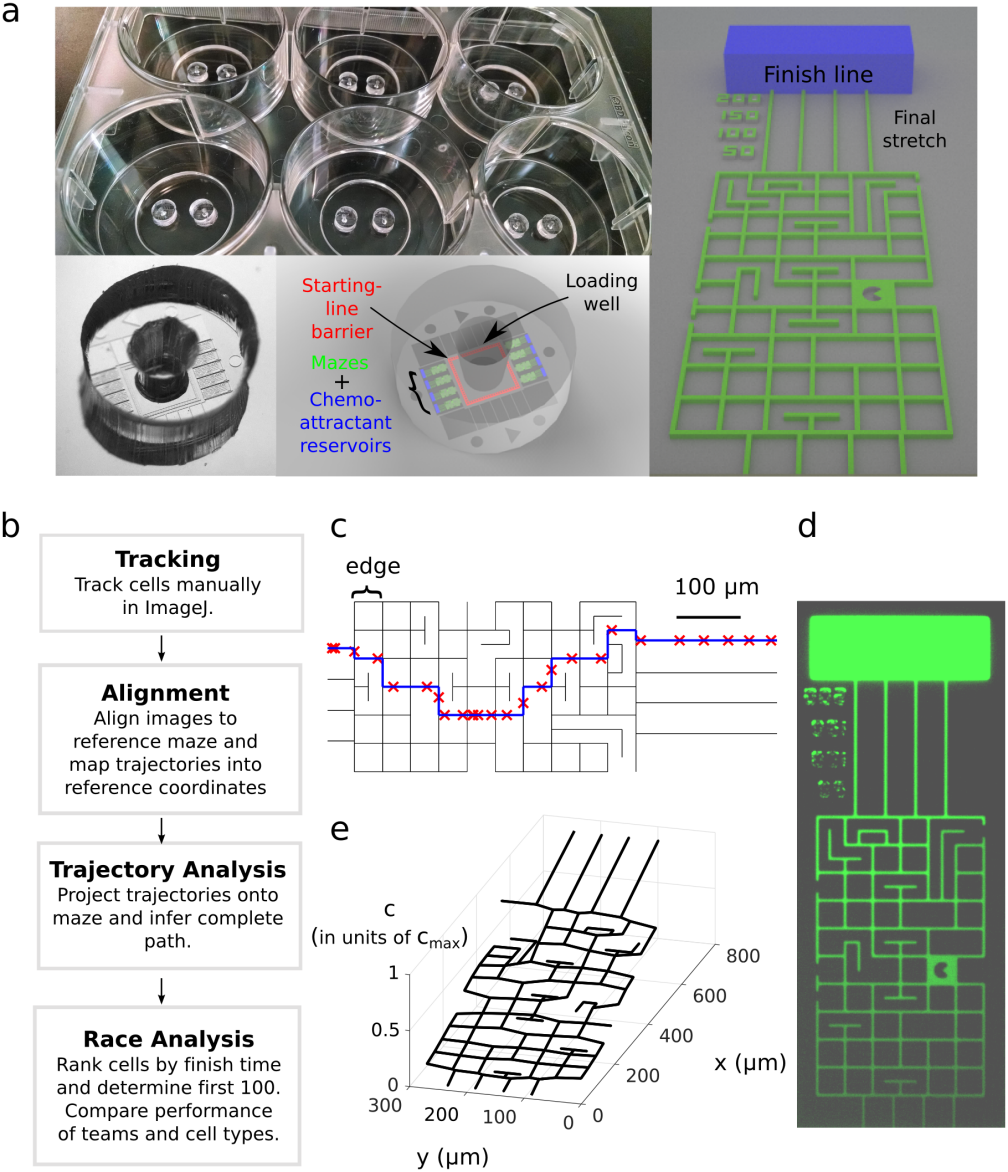
(a) Pictures and sketches of the microfluidic devices and the racecourse. Each well of a 6-well plate contained two devices and each device contained 8 maze racecourses. (b) Flow chart for the analysis of the race. (c) Representation of the maze as a graph with nodes connected by edges, showing a sample cell trajectory projected onto the maze (red crosses) and the inferred path (blue line). (d) Image of the maze loaded with fluorescein. (e) The concentration profile in the maze calculated assuming a constant source of chemoattractant of concentration *c*_*max*_ at the finish line and a sink at the maze entrance.

Each PDMS device was bonded in pairs to a multi-well plate and contained 8 racecourses connected to a central reservoir (Fig 1a). The racecourse was a millimeter-long maze of interconnected, orthogonal channels with a cross-section of 5 × 10 μm (Fig 1b). Each device was designed to have cells loaded in the central reservoir, from which they would enter the mazes through four parallel channels. After traversing the mazes, cells would finish the race through four 220 μm long channels leading to a reservoir with chemoattractant. The starting line was defined by a barrier feature, which was 5 μm tall and 200 μm from the entrance, and would mechanically trap cells during the loading process. The finish line was the entrance to the chemoattractant reservoir. Cells would be guided from the starting line, through the maze, to the finish line by a chemical gradient established by the diffusion of a chemoattractant from the reservoir at the finish line.

### Cell preparation

Axenic *Dictyostelium* cells (see list in Table 1), including a wildtype AX3 strain, were grown in petri dishes with HL5 media (Formedium, UK) to near confluence. Non-axenic *Dictyostelium* cells were grown in petri dishes with bacterial suspension (OD = 2) of *Klebsiella aerogenes* (K.A.) in SorMC buffer (15 mM KH_2_PO_4_, 2 mM Na_2_HPO_4_, 50 mM MgCl_2_, 50 mM CaCl_2_) to near confluence. Cells were harvested by pipetting cells off the dish with developmental buffer (DB, 5 mM Na_2_HPO_4_, 5 mM KH_2_PO_4_, 1 mM CaCl_2_, 2 mM MgCl_2_, pH 6.5), centrifuged at 700g and rinsed twice with DB. A total of ~2x10^7^ cells were plated on non-nutrient KK2 agar dishes to form a dense monolayer. Cells were developed for 5 hours, collected by pipetting with 1 mL DB, and incubated with CellTracker Green CMFDA (Life Technologies) for 30 minutes for fluorescent labeling.

**Table 1:** Participating teams and strategies for enhancing the expected performance of cells in mazes.

Team #	Team members	Cell type	Strategy	Cell tracks and full description
**1**	David Queller, Joan Strassman, Debbie Brock, Tracy Douglas, Susanne DiSalvo, and Suegene Noh, Washington University, St. Louis, US	Dicty	Wild Dicty cells.	https://figshare.com/s/ebf97b9cf877696dc20a
**4**	Guillaume Charras, University College London, UK	HL60	Increase contractility and speed by overexpression of the regulatory light chain of myosin II [60].	https://figshare.com/s/b64e04751618eb05a621
**5**	Natacha Steinckwich-Besancon, National Institutes of Heatlh NIH/NIEHS, US	HL60	Enhance calcium signaling.	
**7**	Terri Bruce, Clemson University, US	Dicty	Increase actin polarization at the leading edge by overexpression of constitutively active Rab8.	https://figshare.com/s/5c60547ee8b6a9757f89
**9**	Robert Insall, Jason King, Peter Thomason, Beatson Institute, UK	Dicty	Eliminate the negative effects of axenic mutations and the associated mutations introduced during axenisation.	https://figshare.com/s/a349669707049b8fef33
**10**	Carsten Beta and Oliver Nagel, U. Potsdam, Germany	Dicty	Decrease cell-substratum adhesion with talin null cells.	
**11**	Jan Faix, Alexander Junemann, Christof Franke and Stefan Bruehmann, Hanover Medical School, Germany	Dicty	Enhanced actin polymerization by overexpression of Rac1A [57].	https://figshare.com/s/7cda43a267f18c65abc3
**12**	Peter van Haastert, Arjan Kortholt, Rama Kataria and Ineke Keizer-Gunnink, U. Groningen, Netherlands	Dicty	Enhance gradient sensing by overexpressing Ric8, a non-receptor GEF for Gα2 [24].	https://figshare.com/s/a1c4630a0f45bfd91192
**14**	Annette Müller-Taubenberger and Matthias Samereier, LMU Munich, Germany	Dicty	Decrease cell-substratum adhesion.	
**15**	Michael Myre, Robert Huber and Susan Cotman, Harvard Medical School, US	Dicty	Precocious development and expression of the chemotactic machinery with CLN3 null cells. The Cln3 gene is involved in Batten disease, a severe childhood neurodegenerative disorder [64].	https://figshare.com/s/d0f92fdd972a3f13a006
**17**	Alan R. Kimmel and Netrapal Meena, National Institutes of Health NIH/NIDDK, US	Dicty	Enhance directionality of chemotaxis by knocking out Gα9 [65, 66].	https://figshare.com/s/e481e604c546c2bb0930
**18**	Robert Kay, Douwe Veltman, MRC Cambridge, UK	Dicty	Enhance the actomyosin cortex in the back of the cell by overexpression of RacGEF in NC4.	https://figshare.com/s/7b70c54846075640e078
**19**	Eric Tschirhart, Sébastien Plancon, University of Luxembourg	HL60	Enhance speed by selection using a Boyden chamber with reference line CCL-240 from ATCC.	
**20**	Peter Devreotes, Kristen Swaney, Thomas Lampert, Johns Hopkins University, US	Dicty	Increase speed by reducing the number of lateral pseudopods by overexpression of CynA [67].	https://figshare.com/s/f4f6c9ab79e85c7ac8f9
Ctrl 1		Dicty	Control Dicty (wildtype AX3 strain)	https://figshare.com/s/cedc88200fda4f2cdd84
Ctrl 2		HL60	Control HL60 (ATCC CCL-240)	https://figshare.com/s/54f82e81e91018e9fddb

Modified and control HL60 cells, (myeloid lymphoma cell line CCL-240 from American Type Culture Collection—ATCC, Manassas, VA) are listed in Table 1. These cells were cultured at 37°C, following the ATCC growth protocol at 100,000 cells/mL density, in Iscove's Modified Dulbecco's Medium (ATCC Catalog No. 30–2005) with fetal bovine serum to a final concentration of 20%. Five days before the race, cells were differentiated by adding 1.3% DMSO to the media. For the race, HL60 cells were incubated with Hoechst dye for 10 minutes at 30 μM concentration.

### Device preparation and loading

Immediately after bonding the PDMS devices to the multi-well plate, the devices were primed with media and chemoattractant. For devices intended for HL60 cells, Iscove’s Modified Dulbecco’s Medium (IMDM, ATCC, Manassas, VA) was used containing 100nM human-fibronectin (Sigma-Aldrich, St. Louis, MO), and 10 nM fMLP (Sigma) as chemoattractant. For devices intended for Dicty cells, DB buffer was used containing 100 nM cAMP (Acros Organics) as chemoattractant and 3 mM caffeine to prevent cell-cell signaling without impairing chemotaxis [22, 23]. For priming, devices were left in a desiccator connected to house vacuum for 15 minutes to ensure the full removal of air bubbles from the chemoattracant chambers. Chemoattractant gradients along the migration channels were established by washing the cell-loading chamber thoroughly using a 1 mL syringe with a 30 G needle containing 1 mL of either IMDM or DB. Flow-through channels having larger cross-sectional area and located on both sides of each maze facilitated this rinsing step. The stability of the gradients in the mazes was analyzed by replacing the chemoattractant with fluorescein (Sigma) and monitoring the fluorescence inside the devices for >6 hours. The molecular weight of fluorescein (332 Da) is comparable to that of cAMP (MW = 329 Da) and fMLP (MW = 438 Da).

### Microscopy and data analysis

For cell loading, 2.5 μL of the cell suspension solution, containing ~20,000 cells, were gently pipetted into the cell loading well using a gel-loading tip. Immediately following cell loading, the plates with *Dictyostelium* and HL60 cells were loaded on two separate fully automated Nikon Eclipse Ti microscopes with environmental chambers. For Dicty cells, the plates were maintained at room temperature (22°C) and 80% humidity throughout the experiment. For HL60 cells, the plates were maintained inside a biochamber heated to 37°C with 5% CO2 and 80% humidity throughout the experiment. Brightfield and fluorescent images (0.1 seconds exposure time) of eight mazes per device per team were taken using multi-point, time-lapse imaging. For the main race, images were taken at the maximal possible rate, which was every 4 min for Dicty and every 5.4 min for HL60, due to the large number of positions imaged. Following the main race, a second race was run to accommodate more Dicty strains (Teams 1, 9 and 18), with images taken every minute. Both races were imaged for 3 hours.

The analysis of the race was divided into four steps, as diagrammed in Fig 1c. Cells were manually tracked in ImageJ and the resulting trajectories were read into MATLAB (The Mathworks, Natick MA) for subsequent analysis. Images were registered at every frame to a reference coordinate system through a custom algorithm that automatically determined the scale, rotation, and translation of the transformation mapping the maze in the input image to the maze in reference coordinates. The trajectories were then projected onto the edges of the maze and the full path of the cell was chosen as the shortest path in the maze going through each sampled point. The times the cell entered and exited the maze were extrapolated using the average speed of the cell over the first and second halves of the trajectory, respectively. Exit times were only defined for cells that were assumed to have finished the race by making it to the final stretch (Fig 1b). The concentration profile in the maze was approximated by the steady-state solution to the diffusion equation for a 1D-approximation of the maze with source and sink of chemoattractant at the finish and entrance of the maze, respectively (Fig 1d). This approximation should be valid for 6 hours, which was twice the span of the race.

## Results

The racecourse for the Dicty World Race 2014 was a millimeter long maze of interconnected, orthogonal channels (Fig 1a). The narrow cross section of the channels, 5 × 10 μm (width × height), was chosen to mimic some of the biomechanical features encountered by neutrophils in tissues and Dicty in the soil. Cells were guided from the starting line, through the maze, and to the finish by a chemical gradient established by the diffusion of a chemoattractant from the reservoir at the finish line (Fig 1d and 1e). Fourteen teams, representing 11 Dicty and 3 HL60 labs, participated in the race and chose genetically and/or pharmacologically enhanced cell lines to compete in the race. The cell lines submitted for the Race and associated strategies are listed in Table 1. Cell lines were shipped to the BioMEMS Resource Center (Charlestown, MA) and the race was run as a one-time event.

**Fig 2:**
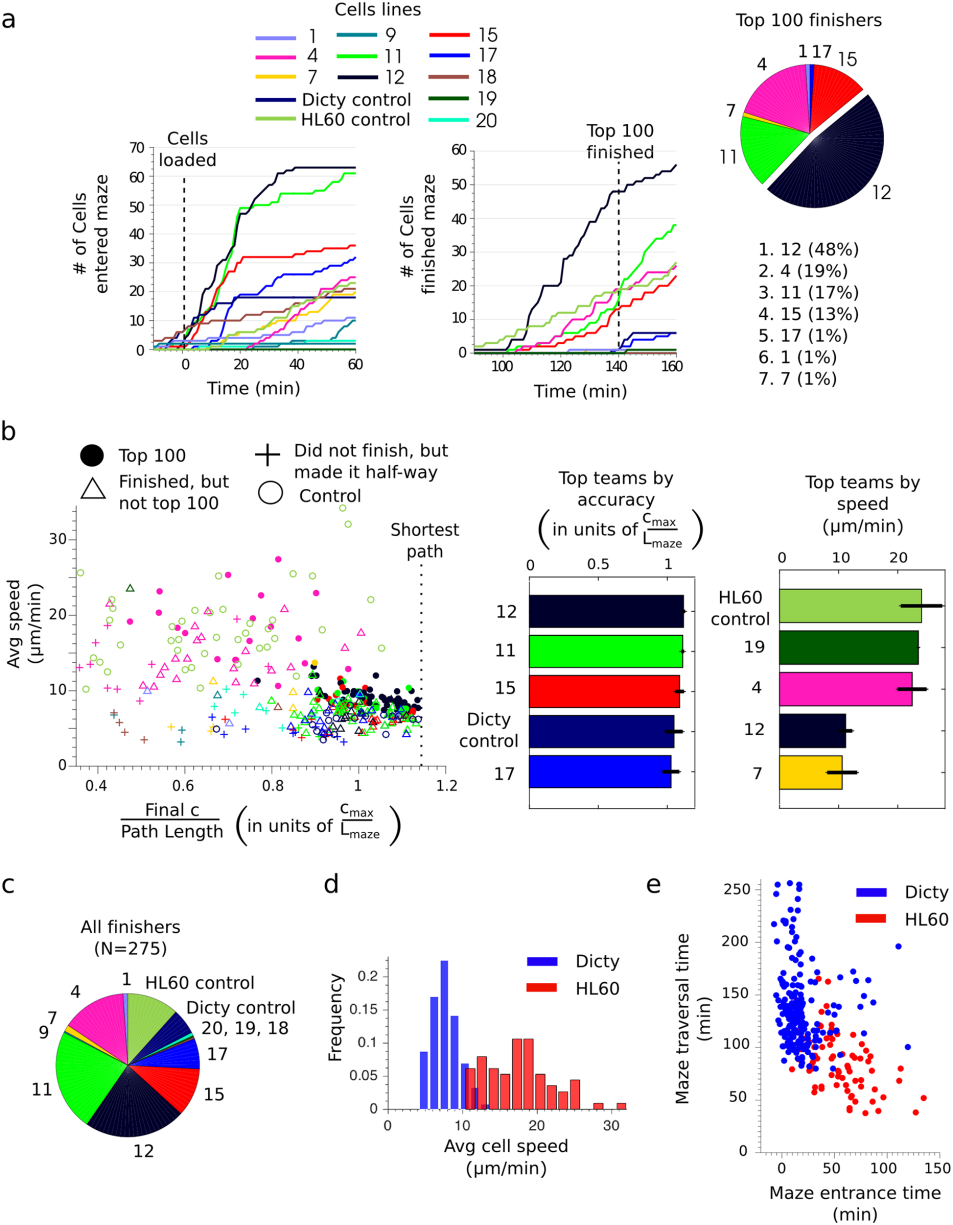
(a) The number of cells having entered (left) or finished (right) the maze as a function of time for each team or control strain. Time 0 corresponds approximately to the time of cell loading. The 100^th^ cell finished the race at 137 minutes. Teams are ranked by their representation in the top 100 cells. The team with the highest representation is the winner. (b) The average speed of a cell versus its chemotactic accuracy, defined as the ratio of the final concentration attained to the path length. Accuracy is measured in units of the characteristic maze gradient *c*_*max*_ / *L*_*maze*_, where *c*_*max*_ is the concentration of chemoattractant at the finish and *L*_*maze*_ = 1 mm. Shown are cells in the top 100 (solid dots, color corresponds to the teams outlined in Fig 2a), the remaining cells that finished the race (triangles), cells that did not finish the race in 3 hours, but made it at least half way (plus signs) and control cells (open circles). Teams were ranked in terms of accuracy and speed by averaging the 10 highest performing cells for each team. Error bars show standard deviations. (c) The representation of each team or control strain in the set of all tracked cells that finished the race. (d) The distributions of cell speed for Dicty (blue) and HL60 (red) cells (e) Comparison of the time taken by each cell to reach the maze entrance to the time taken to traverse the maze.

The results of the race are summarized in Fig 2. A total of 428 cells were observed to finish the race in the 3-hour observation time (individual tracks are available through links in Table 1). Fig 2a shows the cumulative distributions of maze entrance (left) and exit (right) times for each team. Time 0 corresponds approximately to the time when the cells were loaded. The few cells that were estimated to have entered the maze prior to cell loading were likely pushed into the mazes during the loading process. The winner of the race was the team with the highest representation in the first 100 cells to cross the finish line. The race was thus over when the 100^th^ cell crossed the finish line at 140 minutes. Team 12, a Dicty cell line, engineered in the laboratory of A. Kortholt and P. van Haastert, achieved the largest fraction of cells, 48%, in the top 100 and was declared the winner (see S1 Movie and corresponding control, S2 Movie). The runner up with 19% representation was Team 4, an HL60 cell line engineered by the group of G. Charras (see S3 Movie and corresponding control, S4 Movie).

All cells were tracked through the mazes for detailed analysis, except for a minority of cases precluded by a high cell density relative to the frame rate. The performance of these cells was analyzed by comparing their average speed with their chemotactic accuracy, assessed by the ratio of the final concentration reached to the path length (Fig 2b). Final concentrations are in units of *c*_*max*_, the concentration of chemoattractant at the finish (100 nM cAMP for Dicty and 10 nM fMLP for HL60) and path lengths are in units of *L*_*maze*_ = 1 mm, the approximate length of the maze. Shown are cells in the top 100 (solid dots, colors correspond to the legend in Fig 2a), the remaining 127 cells that finished the race after the top 100 (triangles), 43 cells that did not finish the race, but made it at least halfway into the maze (plus signs), and control cells (empty circles). The winning Dicty cells, Team 12, ranked high in chemotactic accuracy, but had significantly lower speed compared to the runner-up HL60 cells, Team 4.

To explore differences between Dicty and HL60 cells, the data from all tracked cells that finished the race (Fig 2c) were pooled together for statistical analysis. A large discrepancy in speed between the cell types was observed, with HL60 cells (v_avg_ = 18 μm/min) moving over twice as fast as Dicty cells (v_avg_ = 8 μm/min) (Fig 2d). To understand how the cell lines had comparable performances despite the large differences in speed, we compared the times taken by cells to enter the mazes to the times spent traversing the mazes (Fig 2e). While HL60 cells were much faster to traverse the mazes, Dicty cells were much quicker to enter the mazes. The race was scored based on the finish times, which are the sum of the maze entrance and traversal times. Interestingly, Team 12 would still have achieved the most cells in the top 100 and won the race if the race was scored on time to traverse the mazes, instead of finish times, though the race would have been much closer between Team 12 and Team 4. Of note, several Dicty cells were already in the maze at the time of the first image, suggesting that the barrier aligning cells at the intended starting line may have been less effective for Dicty cells and may have given some cells a head start.

**Fig 3:**
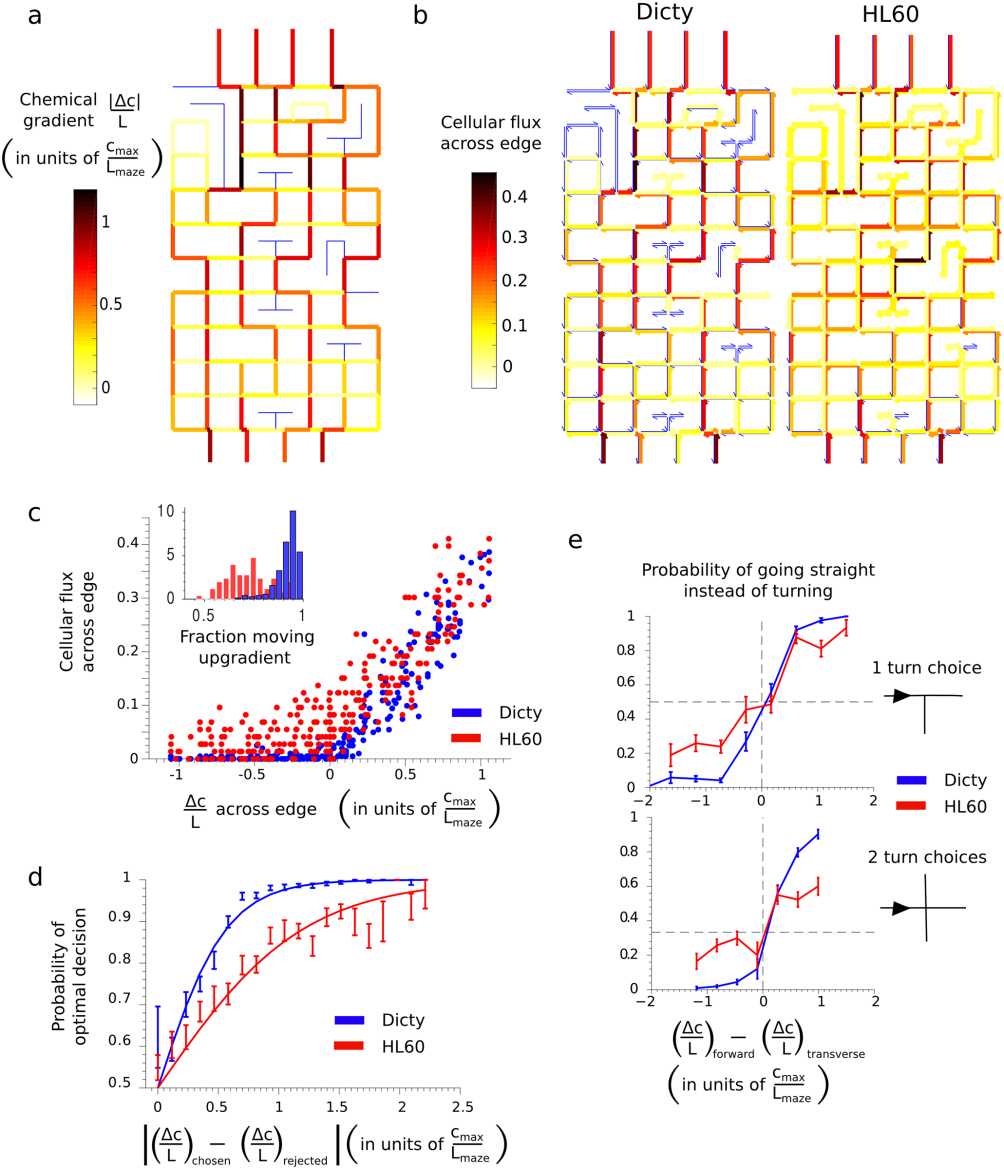
(a) Map of the chemical gradient in the maze. The gradient is measured in units of the characteristic maze gradient *c*_*max*_ / *L*_*maze*_, where *c*_*max*_ is the concentration of chemoattractant at the finish and *L*_*maze*_ = 1 mm. Edges with zero gradient are colored blue. (b) Maps showing cellular flux, the number of cells that took each directed edge in the maze, normalized by the total number of cells analyzed for Dicty (left) and HL60 (right). Edges not traversed by any cells are shown as blue. (c) The cellular flux across an edge as a function of the chemical gradient, Δ*c/L*, across the edge for Dicty (blue) and HL60 (red), where *L* is the length of the edge. The inset shows the fraction of a cell’s trajectory through the maze spent moving up a gradient with Δ*c/L* > 0.25. (d) The probability that a cell chose the better of two choice edges at an intersection as a function of the magnitude of the difference in chemical gradients between the two edges. (e) The probability that cells went straight at a 1-turn (top) or 2-turn (bottom) intersection as a function of the difference in chemical gradients between the forward and transverse edge choices.

To further quantify the chemotactic performance of cells, we generated heat maps representing the magnitude of the chemical gradient (Fig 3a) and the most commonly traversed paths of the maze (Fig 3b). The maze consisted of interconnected, orthogonal segments, or “edges” using terminology from graph theory (Fig 1c). The “cellular flux” across each directed edge was defined as the number of cells that crossed the edge in the specified direction, divided by the total number of cells analyzed. Edges with higher cellular flux were therefore more travelled. If all cells took the exact same path through the maze without retracing their path at any point, the cellular flux along the edges of this path would be 1. Since cells took many different paths through the maze and each cell only traversed a subset of all the edges, the cellular flux was always less than one. The heat map of cellular flux for Dicty cells (Fig 3b) looked very similar to the chemical gradient heat map (Fig 3a), whereas the heat map of cellular flux for HL60 cells was much more uniform indicating a more random choice of path. Quantitatively, the cellular flux across edges in the direction of the gradient was observed to increase similarly for Dicty and HL60 as a function of the chemical gradient Δ*c/L*, where Δ*c* is the concentration drop across the edge of length *L* (Fig 3c). Here and in subsequent analysis chemical gradients are plotted in units of the characteristic maze gradient, *c*_*max*_ */L*_*maze*_, where *c*_*max*_ is the concentration of chemoattractant at the finish and *L*_*maze*_ = 1 mm is the approximate length of the maze. However, the cellular flux across edges in the direction opposite to the gradient (*i*.*e*. downward directed edges pointing away from the finish line in Fig 3b) and in regions of weak gradients (corresponding to yellow edges in Fig 3a) was much higher for HL60 cells than Dicty cells, consistent with HL60 cells taking longer, less direct paths through the maze. Dicty cells spent 92+/-6% (Mean+/-STD) of their trajectories in the maze going up a gradient with Δ*c/L* > 0.25 *c*_*max*_ */L*_*maze*_ versus 72+/-10% for HL60 cells (Fig 3c**, inset)**.

At every junction in the maze, cells were forced to choose among multiple paths. We analyzed these decisions by treating the cell’s choice of path at a junction with N edges as a set of binary decisions in which the cell chose one edge over the N-1 others. Fig 3d shows the probability that cells made the better decision and chose the edge with the larger chemical gradient as a function of the difference in gradient between edges. Dicty cells had a significantly higher probability of making the optimal choice than HL60 cells for small differences in gradient. An analogy can be made between cells traversing the maze and a biased random walk over an energy landscape. Instead of moving downhill in energy, cells are moving uphill in chemoattractant concentration. The probability of making the optimal decision between the chosen and rejected edge choices can be written as $P=\;\frac{1}{1+e^{-\left|{\left(\Delta \text{c}/L\right)}_{\text{chosen}}-{\left(\Delta \text{c}/L\right)}_{\text{rejected}}\right|/T}}$, where Δ*c* is the concentration the cell would gain by taking the edge in units of *c*_*max*_, *L* is the length of the edge in units of *L*_*maze*_, and *T* is an effective temperature, which characterizes the degree of randomness (*i*.*e*. “thermal noise”) during migration. Fitting this function to the data in Fig 3d provided an effective temperature for HL60 cells (T = 0.61), which was twice as large as that for Dicty cells (T = 0.28), consistent with the cellular flux analysis showing HL60 cells followed more random paths than Dicty cells.

To better understand the lower accuracy of HL60 cells, the persistence of cells in the maze was measured by their tendency to go straight, as opposed to turn, at junctions. Persistence could in principle bias cells to go straight even if the gradient cues directed them to turn. The frequency of cells going straight at junctions was measured as a function of the chemical gradient difference between the edge in the forward direction and the edge in the transverse direction (when there were two transverse edges, the maximum gradient of the two was used). The results are shown in Fig 3e for nodes with 1 turn choice (top) and nodes with 2 turn choices (bottom). When unbiased by a chemical gradient, both Dicty and HL60 cells showed no persistence with equal probability of going straight or turning. The lack of persistence at junctions may be due in part to the geometry of the channels in the maze. The narrower, 5-μm width of the channel compared to the 10-μm height, provided more surface area for cell-substratum adhesion along the sidewalls than on the top or bottom. A cell preferentially crawling along a sidewall may be more likely to maintain contact with that sidewall at a junction and thus turn. When biased by the presence of a larger gradient along one of the edges, Dicty cells were significantly more likely to choose the edge with the larger gradient than HL60 cells, in agreement with the findings of Fig 3c.

**Fig 4:**
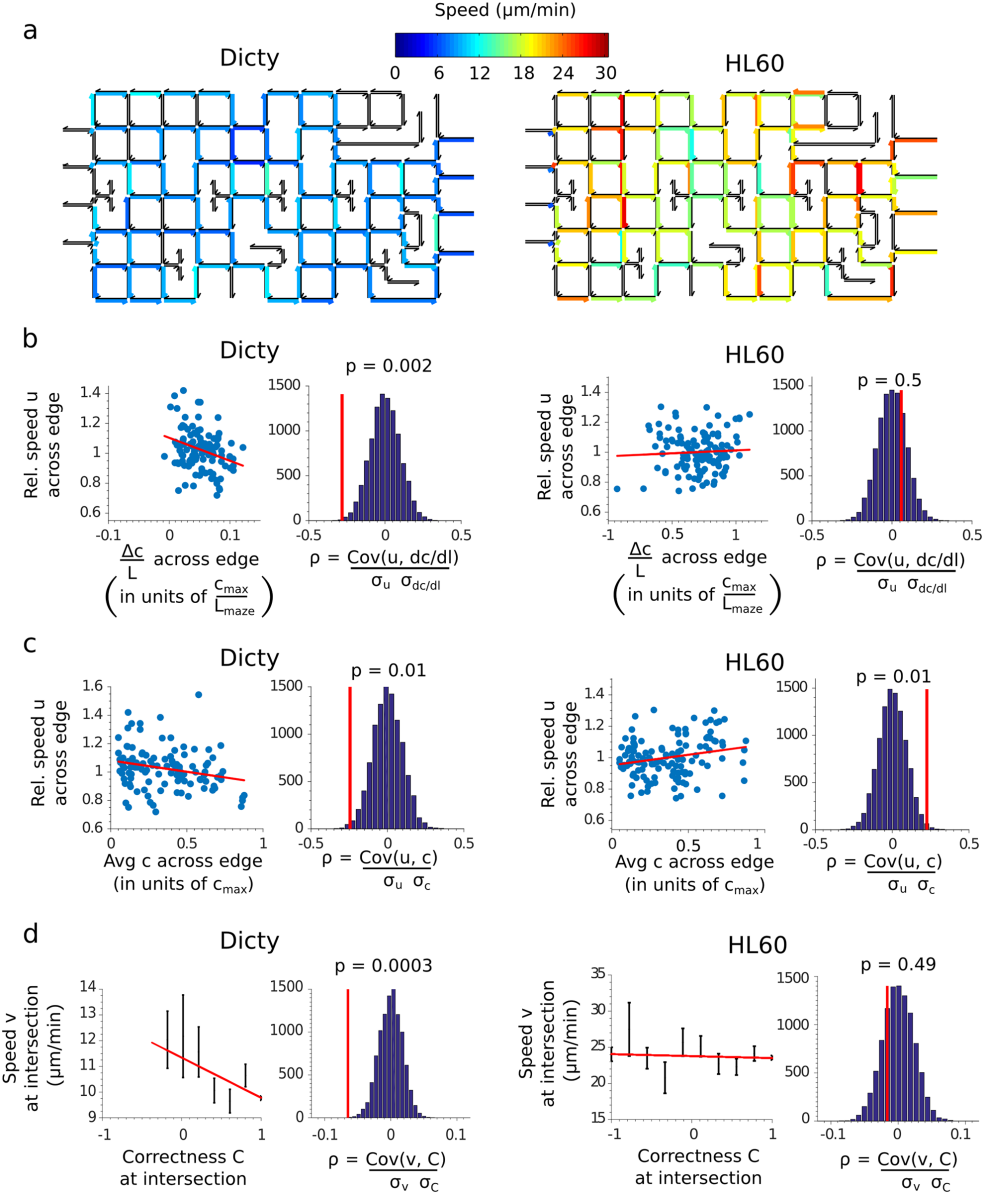
(a) Maps showing the local average speed of cells across each edge in the maze for Dicty (left) and HL60 (right) cells. Edges traveled by less than 5 cells are excluded and shown as black. (b,c) Comparison of the cell speed across an edge, relative to its average value, versus the concentration gradient (b) or average concentration (c) across the edge for Dicty (left) and HL60 (right) cells. Concentrations and lengths are measured in units of *c*_*max*_, the concentration of chemoattractant at the finish, and the maze length *L*_*maze*_ = 1 mm, respectively. Also shown are the correlation coefficients between the variables from the data (red lines) and the distribution of correlation coefficients obtained by 10,000 random resamplings of the data. (d) The cell speed at an intersection versus the correctness C of the cell (see definition in text) at the intersection for Dicty (left) and HL60 (right) cells. Error bars are SEM and only values of C with more than 20 samples are shown. Also shown are the correlation coefficients between the variables from the data (red lines) and the distribution of correlation coefficients obtained by 10,000 random resamplings of the data.

To correlate the chemotactic performance of cells to their speed, directional heat maps representing the average local cell speed were constructed and analyzed (Fig 4a). When the cell speed was normalized to the average for each cell, a strong negative correlation (p = 0.002) between normalized cell speed and chemical gradient across an edge was found for Dicty cells, but not for HL60 cells (Fig 4b). Thus, individual Dicty cells tended to slow down as the gradient increased. Weak correlations were also observed between the normalized cell speed and the average concentration across an edge, with Dicty cells slowing down slightly with increasing concentration (p = 0.01) and HL60 cells speeding up slightly with increasing concentration (p = 0.01) (Fig 4c). Finally, to directly correlate cell speed with chemotactic performance, the performance of cells was rated by their choice of path at each junction. The correctness C of a choice was defined by decomposing the cell’s decision to take 1 of N edges into a series of N-1 binary decisions and giving the cell 1 point for every correct decision and subtracting 1 point for every incorrect decision. The correctness was the weighted average of points from each binary decision, where the weights were proportional to the gradient difference between the two choice edges. Thus, “clearer” decisions involving edges with larger differences in gradient were given larger weights. Interestingly, a strong negative correlation (p = 0.0003) between cell speed and correctness was found for Dicty cells, but no correlation was found for HL60 cells (Fig 4d).

## Discussion

The inaugural Dicty World Race 2014 engaged researchers and the public in a worldwide experiment to test the chemotaxis and motility of model eukaryotic cells, while showcasing the power of microfluidic technology for creative and high-throughput experiments. Researchers were given the novel challenge of engineering Dicty and HL60 cells for enhanced speed and accuracy and applying their knowledge of chemotaxis gained from studies in simple gradients to a complex maze-like racecourse. The race created a fun rivalry between the distantly related cell types and a novel opportunity for a side-by-side comparison of the model systems. The 14 teams that signed up rose to the challenge and came up with a rich set of strategies, representing the diversity of the field. These strategies included enhancing actin polymerization at the front of the cell, increasing contractility of the back of the cell, decreasing cell-substrate adhesion, enhancing chemotactic sensitivity, accelerating development in Dicty and isolating new wild Dicty strains. Many of these strategies were based on studies of chemotaxis in simple linear gradients and it was unknown which strategy would prove optimal in the complex maze racecourse.

Ultimately, the old saying of 'slow and steady wins the race' held true. The winning cells were not the fastest cell type, but excelled in finding the shortest paths through the maze. These *Dictyostelium* cells were engineered with enhanced activity of Ric8, a non-receptor GEF that reactivates heterotrimeric G-proteins [24], resulting in amplification and extension of heterotrimeric G-protein signaling. *Dictyostelium* cells overexpressing this protein show normal chemotaxis in steep gradients of chemoattractant, but have increased directionality in shallow gradients compared to wild-type cells, although with a slight decrease in speed. This increased sensitivity likely played a role in getting cells from the starting line and into the mazes quickly. The increased G-protein signaling may have also increased the “agility” of cells in the maze because Ric8 OE have an increased number of small protrusions at the leading edge that may quicken responses to changing chemical cues. In contrast to the winners, the HL60 cells in second place were among the fastest, but far from the most efficient. These cells were engineered for speed with enhanced contractility through the constitutive overexpression of the regulatory light chain of myosin [25–27]. These striking differences in speed and chemotactic accuracy between the top two teams were representative of the model cell lines as a whole. HL60 cells moved more than twice as fast as Dicty cells, while Dicty cells were far more likely to follow the shortest, directed paths. By quantitatively analyzing cell trajectories as biased random walks, we found that HL60 cells had twice the effective temperature, a measure of randomness analogous to thermal energy, as Dicty cells. Interestingly, the speed of HL60 cells was found to increase slightly with concentration, hinting at an unexpected and potentially important role for chemokinesis and transient pseudo-chemotaxis [28] in getting the fast- and more-randomly moving HL60 cells to the finish line.

Chemotaxis in both cell types involves an interplay between directional sensing, the sampling of the spatial gradient through receptor occupancy, and persistence, the tendency to continue movement in the same direction [29, 30]. While persistence amplifies directional sensing in cells aligned with the gradient and enhances chemotaxis in simple gradients [31] it may interfere with directional sensing in mazes, where the gradient changes direction often. In mazes, agility is important and cells must detect and respond to changes in the gradient direction in the limited time spent at maze junctions. For the current maze we estimate that the time periods for sampling the chemical cues at junctions are ~40 s for Dicty and ~ 20 s for HL60 (from the 5 μm channel width and v_avg_ = 8 μm/min for Dicty and v_avg_ = 18 μm/min for HL60). For Dicty cells, which typically extend ~2 pseudopods per minute, the decision on the path at junctions was made with only one or two pseudopods. The winning team speculates that Ric8^OE^ cells may have an increased rate of sampling the gradient, helping to better bias every pseudopod and giving cells added flexibility to change direction. Similarly, the larger sampling times of Dicty cells versus HL60 cells at junctions due to their twice-as-low speed, may account in part for their more directed and less random paths through the maze. There was a negative correlation between the local cell speed of Dicty cells and their ability to make optimal decisions, hinting that these cells might slow down to make better decisions. Interestingly, there was no such correlation for HL60 cells despite a relatively wide spread in cell speed, although our analysis may be limited by a low time resolution relative to cell speed. The lower chemotactic accuracy of HL60 cells is consistent with previous microfluidic studies [32, 33]. However, another factor may be greater occlusion of the channels by HL60 cells, which blocks diffusion of the chemoattractant and interferes with the chemical gradient [34]. We recommend further studies to clarify the tradeoffs between cell speed and chemotactic accuracy in maze-like environments.

The dramatic difference in speed between HL60 and Dicty cells was unexpected because they have similar migration speeds on commonly studied 2D surfaces [33–36]. While previous work had shown that neutrophils and HL60 cells speed up in confined environments [37], our results show that Dicty cells do not speed up under the conditions tested. This may be due to decreased confinement of Dicty cells due to their slightly smaller size (~7–11 μm diameter for Dicty cells [38, 39], and ~12–16 μm diameter for HL60 cells [40, 41]). However, preliminary attempts to increase the confinement of Dicty cells with narrower channels (6 × 6 μm) were abandoned due to frequent lysis of Dicty cells, which notably did not occur with HL60 cells. An important factor may be cell-substratum adhesion, as the channels were coated with fibronectin for HL60 cells and left uncoated for Dicty cells because they adhere well to naked glass. Strong adhesion is not necessary for movement of neutrophils in tissues [42] and may adversely affect cell speed. Dicty teams 10 and 14 tried to increase cell speed by decreasing cell-substratum adhesion, but this strategy was not tested because these cells did not reach the mazes.

Neutrophils squeeze through narrow pores in blood vessels and the extracellular matrix to reach sites of infection. Dicty cells also move in confined environments as part of forming a multicellular structure during late development. Cell movement switches under conditions of increasing mechanical confinement from being driven by pseudopods via actin polymerization to being driven by blebs [43], which rely on myosin II-based contractility [25–27]. Increasing cell speed by enhancing contractility was the strategy employed by the runner-up HL60 Team 4. One Dicty strain, from Team 18, was also designed for enhanced contractility, but the performance of these cells is unclear due to problems in obtaining sufficient cells on the race day. Mechanical flexibility is also important for cell movement in confinement, with increased rigidity of the cell cortex leading to impaired bleb formation and lowered cell speed [44]. Neutrophils and differentiated HL60 cells have both high compliance on short time scales for squeezing through pores and low viscosity on long time scales for efficient migration in tissues [41]. We recommend further studies comparing the mechanical properties of Dicty and HL60 cells to understand the striking differences in cell speed in the mazes.

While the first Race has mainly uncovered large differences between the model cell types, as opposed to among the engineered cell lines, a key goal of future races will be in evaluating and translating racing strategies into therapeutic strategies for human neutrophils. This general approach is supported by strong conservation of molecular mechanisms of chemotaxis between *Dictyostelium* and neutrophils [18]. Both systems sense chemoattractants with G-protein-coupled receptors [45–48] that activate heterotrimeric G proteins [49–52]. The winning cells, overexpressing the non-receptor GEF Ric8 for G±, highlight the role of non-canonical pathways involving G±, which have recently been shown to play important roles in directional sensing in both systems [24, 53]. The network regulating the assembly of actin in protruding lamellipodia/pseudopodia is also shared by both systems. The conserved Arp2/3 complex mediates branching of actin filaments and is activated by the SCAR/WAVE and WASP nucleating complexes, which are in turn activated by the Rac family of Rho GTPases [54–56]. The third-placed team, Team 11, submitted Dicty cells overexpressing the wildtype Rac1A protein to enhance actin polymerization [57, 58]. Finally, myosin II plays similar roles in generating contractility and mediating rear retraction in both systems. Whereas myosin II is regulated by phosphorylation of both its heavy-chain and regulatory-light-chain subunits in Dicty, the latter dominates in neutrophils through the RhoA/p160ROCK/myosin II pathway [18] and during adhesion-independent migration in confined spaces [27, 59]. The second-placed team, Team 4, submitted HL60 cells overexpressing the regulatory light chain to enhance actomyosin contractility [60].

The narrow outcome of the race between Dicty and HL60 cells is consistent with there being multiple “best” strategies for the complex racecourse. The ability to start moving soon after settling at the starting line, chemotactic sensitivity to weak gradients and fast “2D” migration were all needed to reach the maze entrance. In the mazes, fast migration in narrow channels as well as chemotactic agility and dynamic range were needed to quickly and efficiently reach the finish. The “Dicty” strategy focuses on accurate sensing of the spatial gradients in the maze to guide cells along the shortest paths. In contrast, the “HL60” strategy focuses on sheer speed, at the possible expense of decreased directionality and longer paths, and may involve chemokinesis. Thus, the race leaves many questions: Is there a tradeoff between cell speed and chemotactic accuracy, as suggested by the opposite behaviors of Dicty and HL60 cells? If so, which is more important to neutrophils in fighting infection? What limited the speed of Dicty cells to be less than half that of HL60 cells in narrow channels? Interestingly, even the fastest moving amoeboid cells with speeds of 30 μm/min pale in comparison to that of some of the invading pathogens, such as the parasites causing malaria and toxoplasmosis, with speeds of 1–10 μm/s [61], and marine bacteria, with speeds faster than 100 μm/s [62]. What sets the upper limit to amoeboid speed? Our hope is that future races will continue to explore these questions and give insight into controlling neutrophil behavior in health and disease.

### Future Prospects

While the scientific potential of the race to learn about cell migration is clear, a number of issues need to be optimized for future races. The limitations of an N = 1 experiment were shared with those of previous cell races [63] and were handled in part by relying on previous works validating the robustness and reproducibility of microfluidic cell-migration assays [37] and by racing control strains alongside the competing cell lines. Another challenge for the 2014 race came from the complex logistics of handling a large number of cell lines in parallel. Racing many cell lines at once on a single microscope strained the cell loading process and led to delays between cell loading and imaging, which precluded verifications that all cells began at the intended starting line. Moreover, imaging many cell lines involved scanning many positions and required a significant time delay between subsequent images, which limited detailed analysis of cell behavior in the mazes. Another problem, affecting Teams 1, 5, 9, 10, 14, 18, and 20, was not having enough cells on the race day due to issues with shipping and growing cells in the days leading up to the race, which left the strategies underlying these cells untested. A potential way to overcome these problems in the future will be to have the teams run the race with their cells in their own labs and then collect the results on the race day. This would have the advantage of getting the teams more involved, but the disadvantages of requiring sophisticated microscopes in the labs of participants. Finally, the 2014 'Pac-man'-like racecourse was designed with a focus on visual appeal, and while there were clear differences between Dicty and HL60 strains in terms of cell speed and choice of path, the differences among the cell lines for each model were less apparent. Future mazes should be designed and validated with the goal of maximizing discrimination power.

In summary, the Dicty World Race 2014 demonstrated the feasibility of a fun-spirited competition to bring together a diverse research community and compare cell motility and chemotaxis on a large-scale. Such studies may offer important new insights in complex fields like cell motility. By employing new technologies, future races can creatively explore additional topics like adhesion or phagocytosis. We hope our successes and pitfalls offer valuable learning experiences for these future endeavors.

## Supporting Information

S1 Movie Movie of winning Dicty cells from Team 12. The winning Dicty cells from Team 12 are shown traversing the maze racecourse. Time is shown in minutes. Cells were labeled with CellTracker^™^ Green (Life Technologies) and the fluorescence channel is falsely colored red.
(AVI)

S2 Movie Movie of control Dicty cells. Control wildtype axenic Dicty cells are shown traversing the maze racecourse. Time is shown in minutes. Cells were labeled with CellTracker^™^ Green (Life Technologies) and the fluorescence channel is falsely colored red.
(AVI)

S3 Movie Movie of runner-up HL60 cells from Team 4. The runner-up HL60 cells from Team 4 are shown traversing the maze racecourse. Time is shown in minutes. Cells were labeled with CellTracker^™^ Green (Life Technologies) and the fluorescence channel is shown in green.
(AVI)

S4 Movie Movie of control HL60 cells. Control HL60 cells are shown traversing the maze racecourse. Time is shown in minutes. Cells were labeled with CellTracker^™^ Green (Life Technologies) and the fluorescence channel is shown in green.
(AVI)
